# Effects of simultaneous and sequential cofermentation of *Wickerhamomyces anomalus* and *Saccharomyces cerevisiae* on physicochemical and flavor properties of rice wine

**DOI:** 10.1002/fsn3.1899

**Published:** 2020-11-26

**Authors:** Lihua Chen, Dongna Li, Lixia Ren, Shiqing Song, Xia Ma, Yuzhi Rong

**Affiliations:** ^1^ School of Perfume and Aroma Technology Shanghai Institute of Technology Shanghai China

**Keywords:** cofermentation, flavor characteristics, rice wine, *Saccharomyces cerevisiae*, *Wickerhamomyces anomalus*

## Abstract

Microorganism species and inoculation fermentation methods have great influence on physicochemical and flavor properties of rice wine. Thus, this work investigated microbial interactions and physicochemical and aroma changes of rice wine through different inoculation strategies of *Wickerhamomyces anomalus (W. anomalus)* and *Saccharomyces cerevisiae* (*S. cerevisiae)*. The results underlined that inoculation strategies and *non‐Saccharomyces* yeasts all affected the volatile acidity, total acidity, and alcohol content of rice wine. The sequential cofermentation consumed relatively more sugar and resulted in the higher ethanol content, causing reduced thiols and increased alcohols, esters, phenylethyls, and terpenes, which was more conducive to improve rice wine flavor than simultaneous cofermentation. Moreover, simultaneous cofermentation increased fatty aroma of rice wine, while sequential cofermentation increased mellow and cereal‐like flavor. These results confirmed that sequential cofermentation of *S. cerevisiae* and *W. anomalus* was a choice for the future production of rice wine with good flavor and quality.

## Graphical abstract

Rice wine of sequential cofermentation had mellow and cereal‐like aroma. Sequential cofermentation was more potential to improve rice wine flavor and quality.

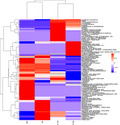

**Abbreviations**
DVB/CAR/PDMS: divinylbenzene/carboxen/polydimethylsiloxane
GC‐MS: gas chromatography‐mass spectrometry
HS‐SPME: headspace solid‐phase microextraction
PCA: principal component analysis
*S. cerevisiae*: Saccharomyces cerevisiae
*W. anomalus*: Wickerhamomyces anomalus

## 1 INTRODUCTION

Rice wine is one of the oldest low‐alcohol brewing wines and popular around the world due to its intense‐rich mellow taste and distinct aroma (Jiang, Mu, Wei, Mu, & Zhao, 2020; Liu et al., 2015; Park, Liu, Park, & Ni, 2016; Yang, Xia, Wang, Yu, & Ai, 2017). Presently, rice wine brewing in the world is based on koji as the natural starter in an open environment (Sun, Liu, & Wang, 2020). The use of koji for fermentation has a long production cycle, which is greatly affected by climate and temperature. Since the quality of mixed bacteria is unstable and potential contamination seriously affects the flavor characteristic of rice wine, especially the sour and spicy taste, many researchers begin to use main microorganisms in koji for fermentation (Lai, Cheng, Lai, Lai, & Ishaq, 2019; Wei, Wang, Zhang, Yuan, & Yue, 2019; Yang et al., 2017). The saccharified rice solution has high monosaccharide content and improved flavor, which has been widely applied in the food and beverage industry. However, few reports were involved in the changes of main functional microorganisms and flavor substances for saccharified rice solution during fermentation.

Fungi molds and yeasts are used as main starter in rice wine, which are responsible for starch degradation and alcohol fermentation, respectively (Sanoppa, Huang, & Wu, 2019; Yang et al., 2017). Meanwhile, wine industrial fermentation tends to use *S. cerevisiae* to ensure the smooth progress of wine fermentation, but that reduces the flavor diversity of wine to some extent (Krogerus, Magalhães, Vidgren, & Gibson, 2017). Recently, research has found that the microbial and brewing characteristics of *non‐Saccharomyces* impact on wine flavor positively (Ciani et al., 2016; Kutyna, Varela, Henschke, Chambers, & Stanley, 2010; Varela, Sengler, Solomon, & Curtin, 2016). It can synthesize many kinds of enzymes and transform the precursor materials into ester, acid, higher alcohol, and other flavor substances, while causing weak alcohol resistance, low fermentation power, and high yield of acetic acid (Ciani et al., 2016). It has been reported that mixed mold cultures can influence flavor compounds in the fermentation process of rice wine production (Liu, Yang, et al., 2019; Yang et al., 2019).

Presently, mixed fermentation of different yeast strains was used in rice wine brewing. Among them, simultaneous cofermentation means that *non‐Saccharomyces* yeasts and *S. cerevisiae* are inoculated at the same time, while sequential fermentation means that *S cerevisiae* is inoculated 1–3 days later after *non‐Saccharomyces* yeasts are inoculated (Shi, Wang, Chen, & Zhang, 2019). Acidity and astringency were the lowest in mixed co‐inoculations, mouthfeel and bitterness were the lowest in *S. cerevisiae* wines, and tasters were preferred to mixed co‐inoculated wines (Minnaar, du Plessis, Jolly, van der Rijst, & du Toit, 2019). The contents of alcohols were significantly decreased by cofermentation of *S. cerevisiae* with *Torulaspora delbrueckii*, but the contents of esters were increased (Liu, Laaksonen, & Yang, 2019). Furthermore, the sequential fermentation of *Hanseniaspora uvarum* and *S. cerevisiae* improved the contents of medium‐chain fatty acid ethyl ester compared with their simultaneous cofermentation (Hu, Jin, Mei, Li, & Tao, 2018). Previous studies have reported that *S. cerevisiae* and *non‐Saccharomyces* did not coexist passively. Instead, they showed interesting interactions that may affect quality of wine (Lencioni et al., 2016). Due to its specific winemaking properties, it may have an additive effect on the flavor and aroma of rice wine. For example, Yang et al. (Yang et al., 2017) studied the volatile compounds of Chinese rice wine fermented by *S. cerevisiae* FC 15 and *S. cerevisiae* BR 30, finding that mixed fermentation rice wine has been highly scored in the overall sense, which indicated that the flavor characteristic of Chinese rice wine can be adjusted by the combination of yeast fermentation. Previous studies pointed that *W. anomalus* was the main strain producing ethyl acetate, which made a special contribution to the Baijiu flavor and quality (Fan et al., 2019). Our previous research also found that *W. anomalus* fermentation produced a large amount of esters and alcohols, which had a strong fruit flavor (Chen, Ren, Li, & Ma, 2020). However, the effects of mixed fermentation of *W. anomalus* and *S. cerevisiae* on aroma and chemical components of rice wine have not been reported.

Thus, this study focused on evaluating the effects of simultaneous and sequential cofermentation of *W. anomalus* with *S. cerevisiae* on aroma, microbial interactions, and physicochemical changes of rice wine through different inoculation strategies. Principal component analysis (PCA) was used to evaluate the influence of inoculum type and inoculation method on volatile compound profile of rice wine. Our study was expected to provide a new starter culture and inoculation method for the rice wine production.

## 2 MATERIALS AND METHODS

### 2.1 Strains and media

**Figure 1:**
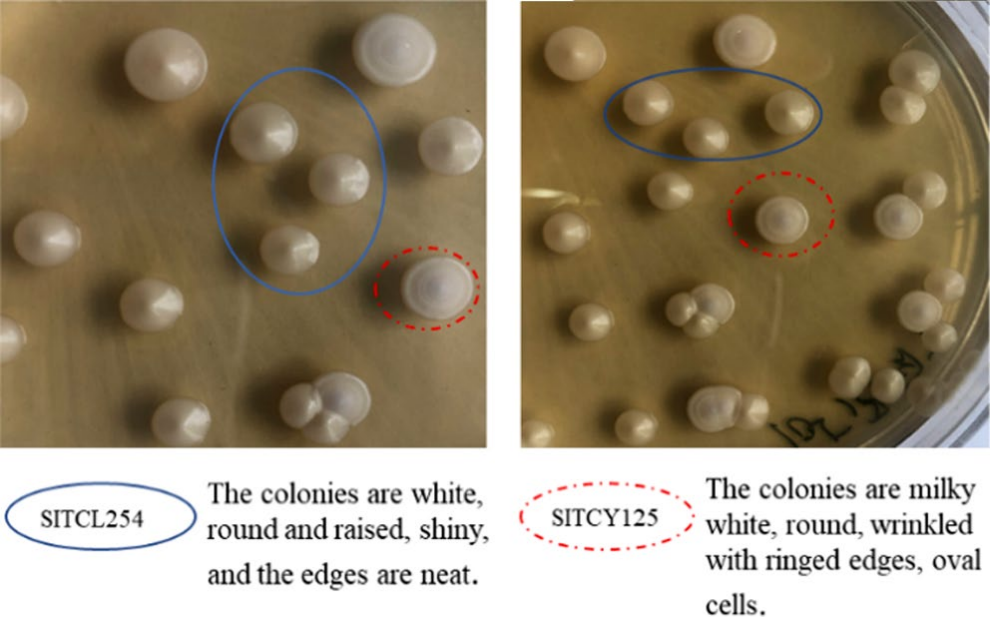
Colony morphology of selected strains SITCL254 and SITCY125 on WLN medium

*S. cerevisiae* (SITCL254) and *non‐Saccharomyces* yeasts (SITCY125) with high fermentability and fragrance production had been isolated from Kijo of Ningbo in Zhejiang and Chongming in Shanghai, respectively. Identification was corroborated by sequencing the D1/D2 variable domains of the 26S rRNA, and their colony morphology is shown in Figure 1.

Glutinous rice was purchased from Chongming. All chemicals and reagents were purchased at Tansoole. 2‐Octanol standards of chromatographic grade were purchased from Sigma‐Aldrich.

### 2.2 Saccharification of rice

The glutinous rice was completely grinded to powder, passed through a 60‐mesh sieve, and stored at −20°C until used. Fifty grams of glutinous rice flour was mixed with distilled water at a certain ratio (1:8 w/v), followed by soaking in a 90°C water bath for 15 min for starch gelatinization. Then, the rice was cooled down to room temperature, and amylase was added (1000 U/g, 0.16%, rice) to water bath at 80°C for 45 min, cooling to room temperature; glucoamylase was added (5000 U/g, 4.8%, rice), bathed at 60°C for 6 hr, and autoclaved at 121°C for 30 min.

### 2.3 Fermentation conditions

Four types of rice wine were prepared as follows: (a) inoculated at 5.05 × 10^6^ CFU/mL SITCL254, (b) inoculated at 5.05 × 10^6^ CFU/mL SITCY125, (c) co‐inoculated at 5 × 10^4^ CFU/mL SITCL254, and 5 × 10^6^ CFU/mL SITCY125, and (d) inoculated at 5 × 10^6^ CFU/mL SITCY125, followed by sequential inoculation of 5 × 10^4^ CFU/mL SITCL254. Fermentations were done in triplicate at 28°C under static conditions. Sampling was carried out every 12 hr to analyze microbial colony count, reducing sugar content, and pH until the end of fermentation. Starter cultures of all yeast strains were grown YPDA liquid medium at 28°C for 24 hr and 120 r/min and were used to start the rice wine fermentation.

### 2.4 Measurement of physiochemical properties

Rice wine samples were clarified and centrifuged at 8,000 rpm for 8 min and then stored at −4°C. The 3,5‐dinitrosalicylic acid (DNS) colorimetric method was used to determine the reducing sugar in the rice wine. WLN medium was used to distinguish the SITCY125 from SITCL254 according to the different color and size of their colonies on plates. Changes in pH were monitored using a pH meter (Mettler Toledo). Alcohol, total acidity, and volatile acidity were determined through methods recommended by Agricultural Industry Standard of the People's Republic of China (NY/T 1885–2017). Total acidity was expressed as lactic acid (g/L), and volatile acidity was expressed as acetic acid (g/L).

### 2.5 Analysis of the volatile compounds by HS‐SPME/GC‐MS

Volatile compounds were identified and quantified as described by Yu et al. (Yu, Xie, Xie, Ai, & Tian, 2019), with slight modifications. The volatile compounds were extracted by headspace solid‐phase microextraction with 50/30 μm DVB/CAR/PDMS fiber (Supelco, Bellefonte, PA, USA) and analyzed using gas chromatography‐mass spectrometry (GC‐MS). Agilent 7,890 gas chromatograph with a HP‐INNOWax column (30 m × 0.25 mm × 0.25 μm, Agilent) coupled to an Agilent 7,890 mass spectrometer was used. 4 ml rice wine samples, 1.5 g NaCl, and internal standard (2‐octanol, 1,760 μg/L) were held in the 20 ml headspace bottle, which was stirred by a magnetic bar in the 50°C water bath for 15 min. After that, the fiber was exposed to the sample headspace for 30 min and immediately followed by desorption of the fiber in the gas chromatography injector at 250°C for 5 min. The GC was operated at the following conditions: initial temperature of 40°C increased to 100°C at 3°C/min and then to 230°C at 10°C /min, a temperature at which it was maintained for 8 min. The injector and detector temperature were all set at 250°C. The flow rate of the carrier gas (helium, 99.999%) was 1 ml/min. The mass spectrometer was operated in electron impact ionization mode at 70 eV, and ion source temperature was 230°C. Compounds were identified by comparing their retention time and MS spectra with their standard compounds, and other compounds were identified by comparing the MS fragmentation patterns which were obtained from database NIST11.

### 2.6 Comparison of the odor activity of rice wine

Comparison of the aroma quality of different processed rice wine samples by the accumulated odor activity values of various volatile components (i.e., the ratio of the content of aroma components to the olfactory threshold, odor active value, OAV). First of all, the OAVs of the same chemical aroma components were calculated ($\sum_{n=1}^{N}\mathrm{OAV}$). The accumulated value matrix of aroma activity is [X_ij_]. Among them, i represents different chemical categories and j represents different processed samples, and then through normalization (i.e., divided by the maximum value of the corresponding category in different processes, X_i_ max), map to [0, 1] interval, and get the matrix [Y_ij_]. The radar images of Y_ij_ were used to show the changes of odor activity of different chemical aroma components in different processed wine samples, and the quality of aroma was compared.

### 2.7 Statistical analysis

Microbial cell enumeration and physicochemical tests were conducted in triplicate. The results were presented as means ± standard deviation. Significant differences among means were tested by one‐way analysis of variance (ANOVA) using SPSS Statistics Software (IBM, version 21) at *p* < .05, and Duncan test was applied for comparison of means. Data and charts were done by Microsoft Office 2010 and Origin 2018. Principal component analysis (PCA) was performed to reduce the dimensionality of the dataset and show the differences in volatile compounds among the rice wine samples. Hierarchical clustering and heat map visualization of volatile compounds in different rice wine samples were performed with Origin 2018 after the Z‐score standardization.

## 3 RESULTS AND DISCUSSION

### 3.1 Microbial concentration and physiochemical properties of rice wine after different fermentations

According to a previous pure fermentation experiment, *W. anomalus* needs to reach 10^6^ ~ 10^7^ cfu/mL to start fermentation in order to prevent the vigorous propagation of *S. cerevisiae*, and two inoculation concentrations of 5.0 × 10^6^ cfu/mL and 5.0 × 10^4^ cfu/mL were selected, respectively, in this study.

**Figure 2:**
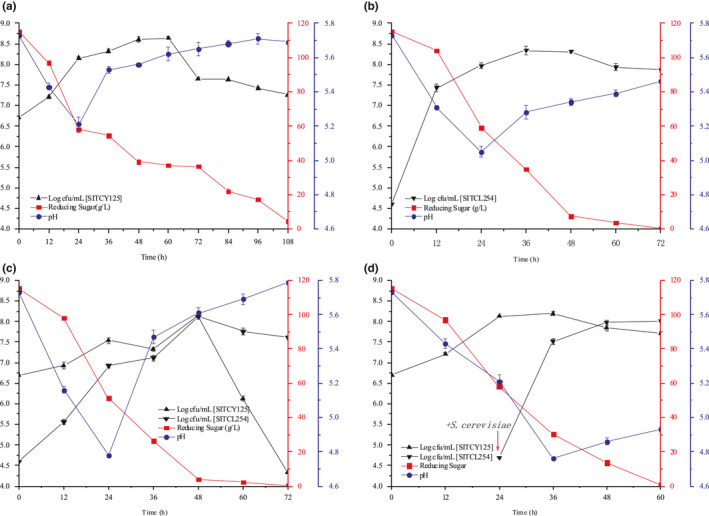
Growth kinetics, reducing sugar, and pH of *W. anomalus* and *S. Cerevisiae* growth during rice wine fermentation with pure culture of *W. anomalus* (a), pure culture of *S. Cerevisiae* (b), simultaneous cofermentation of *W. anomalus*/*S. Cerevisiae* (c), and sequential cofermentation of *W. anomalus*/*S. Cerevisiae* (d)

Figure 2 shows the pure culture fermentation, and *S. cerevisiae* grows faster than *W. anomalus*. Although the initial inoculation amount of *S. cerevisiae* was not high, the cell concentration reached 10^8^ cfu/mL in 24 hr. In the mixed fermentation, the number of *W. anomalus* decreased rapidly after reaching its maximum of 8.51 × 10^7^ cfu/mL. It may be related to the competitive effect of nutrients in the mixed fermentation, the formation concentration of toxic substances (such as ethanol), the population induction of cells, and other factors. In contrast, *S. cerevisiae* maintained a relatively stable rate at a higher order of magnitude (10^7^–10^8^ cfu/mL) until the end of fermentation after reaching its maximum quantity. The results showed that there was obvious competition between the two kinds of yeast. This is consistent with the previous results. *S. cerevisiae* can use the nitrogen source in the substrate faster and more effectively (Liu, Arneborg N, & Toldam‐andersen, 2017), which shows higher fermentation capacity than *non‐Saccharomyces* yeasts in mixed fermentation (Ruiz et al., 2019). Furthermore, in the simultaneous cofermentation, *S. cerevisiae* still kept a high colony number at the end of fermentation. It indicated that *S. cerevisiae* was the dominant yeast, which was similar to the conclusion of Luan (Luan, Zhang, Duan, & Yan, 2018). The maximum biomass of *W. anomalus* and *S. cerevisiae* in sequential cofermentation was significantly higher as compared with those in simultaneous cofermentation. It showed that sequential cofermentation could reduce the inhibition of *S. cerevisiae on W. anomalus* from Figure 2d, which were also observed by Shi (Shi et al., 2019). This may be due to the synergistic effect between *S. cerevisiae* and *W. anomalus* in the sequential cofermentation process, and the relationship between them needs further study.

Sugar is the important substrate of alcohol fermentation. Sequential cofermentations had the fastest sugar consumption than simultaneous cofermentation (Lu, Chua, Huang, Lee, & Liu, 2017; Wei et al., 2020). Compared with the *S. cerevisiae*, the access of *non‐Saccharomyces* yeasts to a certain extent delayed the fermentation process, which is a reflection of the relatively weak fermentation capacity (Domizio et al., 2011). The pH value of fermentation broth shows the same change trend under different strains and their mixed fermentation modes. When the pH value drops to a certain extent, it will rise slowly. Yeasts use sugar in the fermentation broth for growth and reproduction and produce a large number of acid substances, so the pH value in the fermentation broth decreases. In the later stage of fermentation, yeast is in the stage of vigorous alcohol fermentation, and some acids react with alcohols produced in the fermentation process and increase pH value (Kai, Guo, Yin, & Yong, 2018).

### 3.2 Analysis of physicochemical characteristics

Physicochemical characteristics of rice wine samples in different fermentations are shown in Table 1. The mass concentration of reducing sugar in rice wine was lower than 4 g/L, which indicated that rice wine had been fermented completely. The ethanol volume fraction of *S. cerevisiae* pure‐fermented rice wine was the highest, compared with mixed fermented wine. The results showed that *S. cerevisiae* had the strongest ability of reducing sugar transformation. The total acid mass concentration was between 4.32 and 5.02 g/ L. There were some differences among rice wines, and the difference in volatile acid content was the most significant. The concentration of volatile acids in *S. cerevisiae*‐fermented rice wine reached 0.36 g/L, while the mass concentration of volatile acids in simultaneous cofermentation and sequential cofermentation rice wine was only 0.22 and 0.25 g/L. Cofermentations had lower volatile acidity than *S. cerevisiae* fermentation, which was in accordance with results reported by Liu (Liu, Laaksonen, Kortesniemi, Kalpio, & Yang, 2018). The acidity of simultaneous cofermentation was lower than that of sequential cofermentation. These results indicated that fermentation methods, inoculation methods, and *non‐Saccharomyces* yeasts may affect the volatile acidity, total acidity, and alcohol content of rice wine.

**Table 1:** Physicochemical characteristics of rice wine samples in different fermentations

Wines	Residual sugar (g/L)	Alcohol content (%, v/v)	Total acidity (g/L)
P−254	3.60 ± 0.05 ^a^	6.47 ± 0.21 ^c^	4.32 ± 0.24 ^a^
P−125	3.75 ± 0.25 ^b^	4.17 ± 0.19 ^a^	5.02 ± 0.17 ^c^
SiF	2.15 ± 0.10 ^a^	5.49 ± 0.19 ^b^	4.91 ± 0.03 ^b^
SeF	3.96 ± 0.17 ^c^	5.77 ± 0.27 ^b^	4.48 ± 0.08 ^a^

Abbreviations

P‐125, pure fermentation of *W. anomalus*; P‐254, pure fermentation of *S. cerevisiae*; SeF, sequential inoculation fermentation of *W. anomalus/S. cerevisiae*; SiF, simultaneous inoculation fermentation of *W. anomalus/S. cerevisiae*.

Data show average of triplicates ± *SD*. Different letters within columns indicated differences among wine samples determined by the Duncan test at 95% confidence level.

### 3.3 Volatile compounds of rice wine samples in different fermentations

Aroma is one of the most important indicators to measure the quality of rice wine. In this study, ninety‐one aroma compounds were identified in different fermentation wine samples as shown in Table 2. The odor activity value (OAV) is a commonly index used to evaluate the contribution of volatile components of rice wine to the actual aroma. It is widely used in the screening and identification of key odor active compounds in food and can be calculated by the ratio of the concentration to the olfactory threshold of the substance (Wang, Capone, Wilkinson, & Jeffery, 2016). It is generally believed that an OAV greater than 1 indicates that it contributes to the odor, and a larger odor activity value indicates a greater individual contribution of the compound. Compared with *S. cerevisiae* and *W. anomalus* fermentation (376.72 and 766.49 mg/L, respectively), higher content of varietal aroma compounds was detected from cofermentation wine samples. Compared with the corresponding simultaneous cofermentation (870.07 mg/L), varietal aroma compounds (1568.17 mg/L) in sequential cofermentation were higher. These results indicated that the varietal aroma content was affected by the use of *non‐Saccharomyces*, fermentation method, and inoculation strategies. This result was in agreement with a previous study (Wei et al., 2020).

**Table 2:** Volatile compounds of rice wine samples in different fermentations

RT/min	Compounds	Concentration (μg/L)				Odor threshold (μg/L)^a^	OAV^b^	Odors^c^
		P−254	P−125	SiF	SeF			
	Alcohols							
26.3162	Benzyl alcohol	82.47				20,000 [1]	<0.1	Characteristic, Pleasant, Fruity, Pungent, Sweet, Almond, Fatty [1], [2]
8.2285	Ethanol	128,986.95	258,440.60	170,823.17	500,813.58	1,499.85 [2]	>1	Slight, Characteristic, Burning [1]
26.7683	Phenylethyl Alcohol	91,592.49	120,423.34	274,006.79	392,464.54	14,000 [1]	>1	Rose‐like, Bitter, Sweet, Peach, Flowery, Pollen, Perfume [1]. [2], [4]
10.5511	1‐Propanol	424.48	900.02	776.65	1,038.83	5.44 [2]	>1	Alcoholic, Characteristic, Ripe, Fruity [1],[5]
15.3378	1‐Butanol	147.14	280.28		422.40	2.30 [2]	>1	Dry, Burning, Wine [1], [4]
12.1706	2‐Methyl−1‐propanol	3,143.70	6,130.41	6,300.39	8,472.04	34.93 [2]	>1	Penetrating, Wine‐like, Disagreeable [1]
16.2799	1‐Pentanol	70.31	162.77		226.65	0.90 [2]	>1	Characteristic, Fusel‐like, Sweet, Pleasant, Burning [1]
15.3148	3‐Methyl−1‐butanol	56,474.08	100,598.54	133,765.97		784.36 [2]	>1	Fusel oil, Whiskey‐characteristic, Pungent, Repulsive, Alcohol, Nail polish [1], [4]
17.9552	(S)‐(+)−3‐Methyl−1‐pentanol	425.79		323.80		5.91 [2]	>1	
18.4636	1‐Hexanol	1,132.82	3,183.15	3,972.84	3,823.92	14.52[2]	>1	Herbaceous, Woody, Fragrant, Mild, Sweet, Green, Fruity, Aromatic, Green, Floral [1], [2], [4],[5]
20.3423	1‐Heptanol			1,479.21	1,468.10	5.40 [2]	>1	Fragrant, Woody, Oily, Faint, Aromatic, Fatty, Pungent, Spicy [1]
21.9727	1‐Octanol			3,117.34		125.80 [2]	>1	Fresh, Orange‐rose, Sweet, Oily, Herbaceous, Fruity[1], [5]
20.9178	1‐Hexanol, 2‐ethyl‐			5,984.96	9,299.67	25,482.20 [2]	0.1–1	Mild, Oily, Sweet, Floral, Rose, Fatty‐floral, Fruity [1]
23.4125	1‐Nonanol		1576.34	2025.25		45.5 [2]	>1	Rose‐orange, Fatty, Bitter, Orange [1]
31.6347	Glycerin	14,352.14	34,042.33		7,386.85	184.0 [2]	>1	Sweet [1]
23.5399	2‐Furanmethanol	3,556.02	4,388.68			36.66 [2]	>1	Mild, Warm, Oily, Burnt, Cooked sugar [1]
24.1119	L‐.alpha.‐Terpineol	92.45				1.06 [2]	>1	
25.7634	Geraniol	777.35	1524.11	1,620.20	2,783.38	8.54 [2]	>1	Rose‐like. [1]
21.8243	1,6‐Octadien−3‐ol, 3,7‐dimethyl‐				1,382.24	00.22 [2]	>1	Pleasant floral [1]
24.7812	Citronellol			1906.97	1895.65	62.0–2200 [2]	0.1–1	Rose‐like, Bitter, Sweet, Peach‐like [1]
25.0902	4‐Hexen−1‐ol, 5‐methyl−2‐(1‐methylethenyl)‐, (R)‐			1,292.96				
24.3632	3‐(Methylsulfanyl)−1‐propanol	1,296.45	1,560.67	4,913.18	5,238.79	13.79 [2]	>1	Powerful, Sweet, Soup, Meat‐like [1]
20.2405	1‐Octen−3‐ol	78.56	483.09	409.83	456.74	1.83 [2]	>1	Powerful, Sweet, Herbaceous, Reminiscent, Lavender–lavandin, Rose, Hay, Herbaceous [1]
16.3374	3‐Buten−1‐ol, 3‐methyl‐				216.35	547.13 [2]	0.1–1	
21.7569	2,3‐Butanediol	867.19	443.05	532.90		10.84 [2]	>1	
26.1693	Total	303,500.40	534,137.37	611,959.47	938,682.69			
	Esters							
26.1693	9,12,15‐Octadecatrienoic acid, methyl ester, (Z,Z,Z)‐	177.95				5.08 [2]	>1	
23.7515	Pentanoic acid, 4‐methyl‐, methyl ester			8,378.22				Sweet, Pineapple‐like [1]
7.1351	Ethyl Acetate	2,804.28	3,080.44	25,121.52	8,435.98	35.05 [2]	>1	Pleasant, Ethereal, Fruity, Brandy‐like, pineapple, Fruity, Sweet, Fermentation [1], [2], [3].[5]
23.8834	Benzoic acid, ethyl ester	263.46	483.09	955.87		3.76 [2]	>1	Fruity, Floral [1],[4]
18.0457	Hexanoic acid, ethyl ester	4,117.66	6,824.13	9,319.76	20,943.14	42.02 [2]	>1	Powerful, Fruity, Pineapple–banana, Winy [1]
26.2272	Butanoic acid, ethyl ester	705.74				17.21 [2]	>1	Fruity, Pineapple, Sweet, Analogous [1]
23.2161	Decanoic acid, ethyl ester	2,149.76	3,291.08	4,017.05		22.63 [2]	>1	Fruity, Grape (cognac), Oily, Brandy‐like [1]
21.7258	Nonanoic acid, ethyl ester			353.67	523.71	377.0 [2]	0.1–1	Fatty, Oily, Nutty, Fruity, Cognac, Rosy‐fruity [1]
26.4789	Benzenepropanoic acid, ethyl ester	227.87	486.57	616.54		3.93 [2]	>1	Ethereal, Rum, Fruity, Floral [1]
25.3918	2‐Propenoic acid, 3‐phenyl‐, 2‐phenylethyl ester	474.83				10.10 [2]	>1	Sweet, Balsamic, Rose, Plum‐like [1]
30.4816	Hexadecanoic acid, ethyl ester	553.82	4,691.59	1,530.59	3,384.35	5.60 [2]	>1	Mild, Waxy, Sweet [1]
21.7467	Acetic acid, methoxy‐, ethyl ester		1,256.89					
26.3453	Hexanoic acid, 3‐hydroxy‐, ethyl ester			753.94				Fruity [1]
26.3485	Hexanoic acid, pentyl ester	71.62				01.89 [2]	>1	Characteristic, Fruit‐like (banana, pineapple) [1]
12.7541	3‐methyl−1‐butanol acetate	687.51	2,286.61			7.64 [2]	>1	Fruity, Banana, Sweet, Fragrant, Powerful, Bittersweet, Pear, Alcohol, Nail polish [1],[4]
15.3411	1‐Butanol, 3‐methyl‐, formate				230,060.33	149.0 [2]	>1	Plum, Fruity, Black currant, Sweet [1]
26.0133	Propanoic acid, 2‐methyl‐, 2‐methylbutyl ester				1,116.10	14.0 [2]	>1	
27.6999	Formic acid, heptyl ester		1,094.12					Fruity‐floral, Orris‐rose, Sweet, Plum [1]
27.6999	Formic acid, phenyl ester		352.52					
26.3485	Acetic acid, phenyl ester	238.28				3.40 [2]	>1	Offensive [1]
26.6555	Butylated hydroxytoluene	491.76	60,343.11	954.68	5,984.00	5.07 [2]	>1	Faint, Musty, Occasional cresylic‐type [1]
25.6843	Acetic acid, phenethyl ester	8,503.56	12,990.23	23,323.29	65,525.23	94.48 [2]	>1	Floral, Rose, Honey‐like, Sweet, Fruit‐like, Raspberry [1]
26.4684	Propanoic acid, 2,2‐dimethyl‐, 2‐phenylethyl ester			1725.66				
	Total	21,468.10	97,180.38	75,325.13	337,698.50			
	Aldehydes							
21.896	Benzaldehyde	344.19	403.88	812.49	982.17	5.74 [2]	>1	Characteristic, Bitter almond, Burnt sugar [1],[4]
23.6123	Benzeneacetaldehyde			3,056.40	4,649.83	4.0 [2]	>1	Harsh, Green, Hyacinth, Unpleasant, Pungent, Bitter, Flavor, Turning sweet, Fruit‐like [1]
7.8012	Butanal, 3‐methyl‐	92.01				1.53 [2]	>1	Choking, Powerful, Acrid, Pungent, Apple‐like, Fruity, Fatty, Animal, Almond [1]
19.362	Nonanal	185.33	383.86	457.62	393.21	1.93 [2]	>1	Fatty, Orange, Rose, Citrus‐like [1]
20.8056	Furfural	1881.09	2,290.09			20.67 [2]	>1	Characteristic penetrating [1]
20.8073	3‐Furaldehyde				252.41			Sour, [4]
22.5548	5‐Methylfuran−2‐carbaldehyde	865.46	1,334.36			9.31 [2]	>1	Sweet, Spicy, Warm, Caramel‐like [1]
	Total	3,368.09	4,412.19	4,326.52	6,277.62			
	Phenols							
27.6197	Phenol, 2‐methyl‐	233.08	323.80	978.57		2.56 [2]	>1	Musty, Phenolic [1]
27.6936	Phenol			1,010.84		4600–9000 [2]	0.1–1	Sweet [1]
27.4218	Maltol	967.89	1,092.38			12.74 [2]	>1	Caramel–butterscotch, Jam‐like, Fruity, Strawberry [1]
31.2561	2,4‐Bis(1,1‐dimethylethyl)‐phenol	14,120.80		75,470.90	80,726.48	148.64 [2]	>1	
31.265	Phenol, 3,5‐bis(1,1‐dimethylethyl)‐	74,179.39						
30.0983	2‐Methoxy−4‐vinylphenol	1,397.58	3,799.41	4,913.18		19.97 [2]	>1	Powerful, Spicy, Apple, Rum, Roasted peanut [1]
	Total	16,719.35	79,394.98	82,373.50	80,726.48			
	Ketones							
17.7353	1‐hydroxy−2‐propanone	968.76	899.15		295.34	13.46 [2]	>1	
26.002	5,9‐Undecadien−2‐one, 6,10‐dimethyl‐, (E)‐		1,460.57			60.0 [2]	>1	Green, Rosy, Floral, Fresh‐floral, Penetrating. [1]
17.4199	Acetoin	66.41	133.18			0.92 [2]	>1	Bland, Woody, Yogurt, Fatty, Creamy “tub,” Flavor, Butter, Milk, Yogurt, Strawberry [1]
17.1376	2‐Octanone	69.88	92.27			0.86 [2]	>1	Floral, Bitter, Green, Fruity (unripe, apple), Camphoraceous [1]
21.9771	2‐Methyldihydrothiophen−3(2H)‐one	1,057.30			4,017.95	15.10 [2]	>1	
	Total	2,162.35	2,585.16	0.00	4,313.29			
	Acids							
24.306	2‐Amino−6‐methylbenzoic acid				1814.95			
20.5444	Acetic acid	7,085.14	9,857.57	17,989.52	3,756.96	77.86 [2]	>1	Sour, Pungent [4]
22.3588	Propanoic acid, 2‐methyl‐			2,903.46	4,107.24	6,550.60 [2]	0.1–1	Penetrating, Rancid, Butter [1]
25.8683	Hexanoic acid	6,002.66	7,314.18	18,073.16	11,166.13	420.0 [1]	>1	Sickening, Sweaty, Rancid, Sour, Sharp, Pungent, Cheesy, Fatty, Unpleasant [1],[2],[5]
27.0476	Heptanoic acid	1,460.95	2,135.15	4,992.04		20.87 [2]	>1	Disagreeable, Rancid, Sour, Sweat‐like, Fatty [1]
28.2015	Octanoic acid	9,976.23	14,101.76	35,493.93	35,282.42	120.20 [2]	>1	Unpleasant, Burning, Rancid, Cheese, Harsh, Fatty acid [1], [2],[5]
30.9251	*n*‐Decanoic acid	1,284.30	7,802.49	4,236.90	6,847.69	1,000 [1]	>1	Fatty, Unpleasant, Rancid [1], [2]
27.1628	*n*‐Hexadecanoic acid				114,516.75			Characteristic [1]
33.7337	9,12‐Octadecadienoic acid (Z,Z)‐				15,536.08			
29.4811	Nonanoic acid			1,458.90				Fatty, Unpleasant, Cheese, Waxy [1],[5]
	Total	25,809.28	41,211.16	85,147.92	193,028.21			
	Terpenes							
17.9455	1‐Heptene, 6‐methyl‐				1,267.20			
22.4346	Ethene, fluoro‐	237.42				3.30 [2]	>1	
24.722	1‐Tridecene		487.44					
14.792	D‐Limonene	720.93	210.64	270.03		7.28 [2]	>1	Pleasant, Lemon‐like, Camphoraceous, Turpentine‐like [1]
	Total	958.34	698.08	270.03	1,267.20			
	Others							
21.1136	Heptadecane, 8‐methyl‐			609.37				
22.6345	Nonadecane			474.35	460.18			
17.9402	Pentane, 2‐cyclopropyl‐		190.62					
31.9168	Diglycerol		1702.55					
23.0086	Ethanol, 2‐(2‐ethoxyethoxy)‐			738.41		1,600 [2]	0.1–1	
15.6804	Furan, 2‐pentyl‐			235.38	221.50	5.80 [2]	>1	Fruity, Green bean, Metallic, Vegetable [1]
21.4986	1‐(2‐Furanyl)ethanone	134.55	146.23			1.77 [2]	>1	Coffee‐like [1]
33.0359	2,3‐Dihydrobenzofuran	2,172.76	3,156.16	7,207.28	5,491.20	26.18 [2]	>1	
28.5217	Phenol, 3‐(1,1‐dimethylethyl)−4‐methoxy‐	1679.92						
27.5515	Benzothiazole			1,399.16		80.0 [2]	>1	Rose‐like [1]
	Total	2,736.57	6,875.49	10,663.95	6,172.88			
	£	376,722.47	766,494.80	870,066.51	1,568,166.85			

Abbreviations

P‐125, *W. anomalus*; P‐254, *S. cerevisiae* fermentation; RT: retention time; SeF, sequential inoculation fermentation with *W. anomalus* and *S. cerevisiae*; SiF, simultaneous inoculation fermentation with *W. anomalus* and *S. cerevisiae*.

^a^ Odor threshold was obtained from literatures: [1] Shi et al., 2019; [2] 《ODOUR THRESHOLDS》, Published by Oliemans Punter & Partners BV, The Netherlands.

^b^ OAV was calculated by dividing concentration by the odor threshold value of the compound. The scope of OAV was shown but not specific value.

^c^ Odors were obtained from literatures: [1] FENAROLI’S HANDBOOK OF Flavor Ingredients; [2] Shi et al., 2019; [3] Niu et al., 2018; [4] Yu et al., 2019; [5] MO, Xu, & Fan, 2010.

Alcohol is one of the most important component types in the rice wine. Higher alcohols are mainly produced by transamination of amino acids as substrates and reduction of alcohol dehydrogenase. Compared with pure fermentation of *S. cerevisiae* (127.19 mg/L), the content of higher alcohols in cofermentations was significantly increased, which was in agreement with a previous report which pointed that more ethanol was produced in mixed culture fermentation with *S. cerevisiae* and *W. anomalus* fermentation and accumulation of primary metabolites could influence microbial interaction, end‐product flavor, and Baijiu quality (Zha, Sun, Wu, Yin, & Wang, 2018). Moreover, the content of higher alcohols was also higher in the simultaneous cofermentation (294.85 mg/L) than that in sequential cofermentation (414.87 mg/L). It has been reported that when the concentration of higher alcohols exceeds 400 mg/L, they have a negative effect on wine flavor, and the concentration of 300–400 mg/L is acceptable, whereas the optimal level (below 300 mg/L) imparts a pleasant character (Luan et al., 2018). These results indicated that the simultaneous cofermentation of *non‐Saccharomyces* yeasts and *S. cerevisiae* was more conductive to producing appropriate content of higher *non‐Saccharomyces* yeast alcohols in the wine. Interestingly, 3‐methyl‐1‐butanol which was reported to have the nail polish odor (Liu, Yang, et al., 2019) was not detected in sequential cofermentations and relatively abundant especially in the rice wine from the pure fermentation of *S. cerevisiae* and simultaneous cofermentation. The reason may be that *W. anomalus* can provide nutrients for *S. cerevisiae* in the later stage of fermentation, or *W. anomalus* has some enzyme activities, which can provide nutrients for *S. cerevisiae*. Additionally, C_6_ alcohols usually have the characteristics of "plant" and "turf," which have a negative impact on the aroma of wine (Luan et al., 2018). Compared with *S. cerevisiae*, C_6_ alcohol (3‐methyl‐pentanol) was not detected in sequential cofermentation. Sequential cofermentation method can effectively decrease C_6_ alcohol formation. The high alcohol in sequential cofermentation had a similar response to rice as other three ways of fermentation, but their amounts were higher than those of them, especially with phenylethyl alcohol, 1‐propanol, 2‐ethyl‐1‐hexanol and glycerin (contributing to “smoothness,” “sweetness,” and “complexity” notes for wine) (Binati et al., 2020), and the total contents. The increase of 2‐phenylethanol during mixed fermentation seems to be related to the synergistic effect of these two different strains. In pure culture, both of yeasts are producers of low‐2‐phenylethanol, as previously noted for other pairings of yeast species (GOBBI, Comitini, Domizio, Romani, & Lencioni, 2013). This indicated that sequential cofermentation had a relatively strong ability to synthesis higher alcohol.

Esters (including acetate esters and fatty acid ethyl esters) were one of the main products of fermentation, and it is mainly produced by yeast metabolism and esterification reaction in wine, with flower and fruit fragrance (Cao, Wu, & Weng, 2020). Compared with pure fermentation, the content of total esters in sequential cofermentation was higher. Cofermentations significantly enhanced the production of ethyl acetate, hexanoic ethyl ester, and acetic acid phenethyl ester. Interestingly, sequential cofermentation produces significantly higher amounts of hexanoic ethyl ester and acetic acid phenethyl ester than simultaneous co‐inoculation. This may be due to the sequential cofermentation of *non‐Saccharomyces* yeasts and *S. cerevisiae* contributed to the formation of esters, which was also reported by other researchers (Shi et al., 2019; Tristezza et al., 2016; Zhang, Luan, Duan, & Yan, 2018). The mixed fermentation of *W. anomalus* and *S. cerevisiae* not only increased the yield of ethyl acetate, but also increased the content of other flavor substances such as β‐phenethyl alcohol and phenethyl acetate, which provided an opportunity to change the aroma and flavor of liquor (Fan et al., 2019). In addition, it is reported that low concentrations of ethyl acetate (<150 mg/L) will bring fruity and pleasant aromas to wine (Xiao et al., 2015). Since the concentrations of ethyl acetate ranged between 2.80 and 8.44 mg/L in our study, it was likely that the presence of this compound positively affected rice wine quality.

When the concentration of fatty acids is low, they are creamy and cheesy, while when the concentration is too high, they will produce sour and sour taste (Niu et al., 2019). The highest total amounts of fatty acids, decanoic acid, and octanoic acid were produced in pure fermentation of *W. anomalus*, that *W. anomalus* strains produced lower levels of fatty acid (decanoic acid) than *S. cerevisiae*. It was interesting to notice that the octanoic acid in sequential cofermentation was 61.89% lower than that in pure fermentation of *W. anomalus*. This meant that the rice wine aroma is more harmonious and balanced. Likewise, in mixed fermentation, the content of hexanoic acid and octanoic acid is higher than that of pure fermentation. These results indicated the sequential cofermentation of *non‐Saccharomyces* yeasts and *S. cerevisiae* would contribute to the formation of fatty acid in the wine. This conclusion is consistent with the previous results of Ma and Wang (Ma, Yan, Wang, Zhang, & Tao, 2017; Wang, Tao, Wu, An, & Yue, 2017).

Terpenes have strong physiological activity to the human body. Generally, it exists in the form of glycosides, which also contributes to the aroma of wine. The mixed fermentation of *S. cerevisiae* and *W. anomalus* was beneficial to the formation of citronellol (strong smell of rose) (Pratibha et al., 2018), which was not detected in pure fermentation. The content of terpenoids produced by sequential cofermentation was the highest, reaching 7.33 mg/L. Compared with pure fermentation, the content of linalool (lavender), geraniol (rose), and 6‐methyl‐1‐heptene slightly increased after mixed fermentation. Therefore, sequential cofermentation can improve the aroma complexity of rice wine.

2‐Octanone and 2,4‐Bis(1,1‐dimethylethyl)‐phenol were also detected in the rice wine samples, which contributed to the wine body balance. 2,4‐bis(1,1‐dimethylethyl)phenol (medicinal, tobacco and phenolic flavors) was detected in the fermentation process of rice wine, but the content of 2,4‐Bis(1,1‐dimethylethyl)‐phenol in the sequential co‐fermentation rice wine samples was higher than pure fermentation samples.

### 3.4 Cluster heat map of volatile aroma compounds in different fermentations

**Figure 3:**
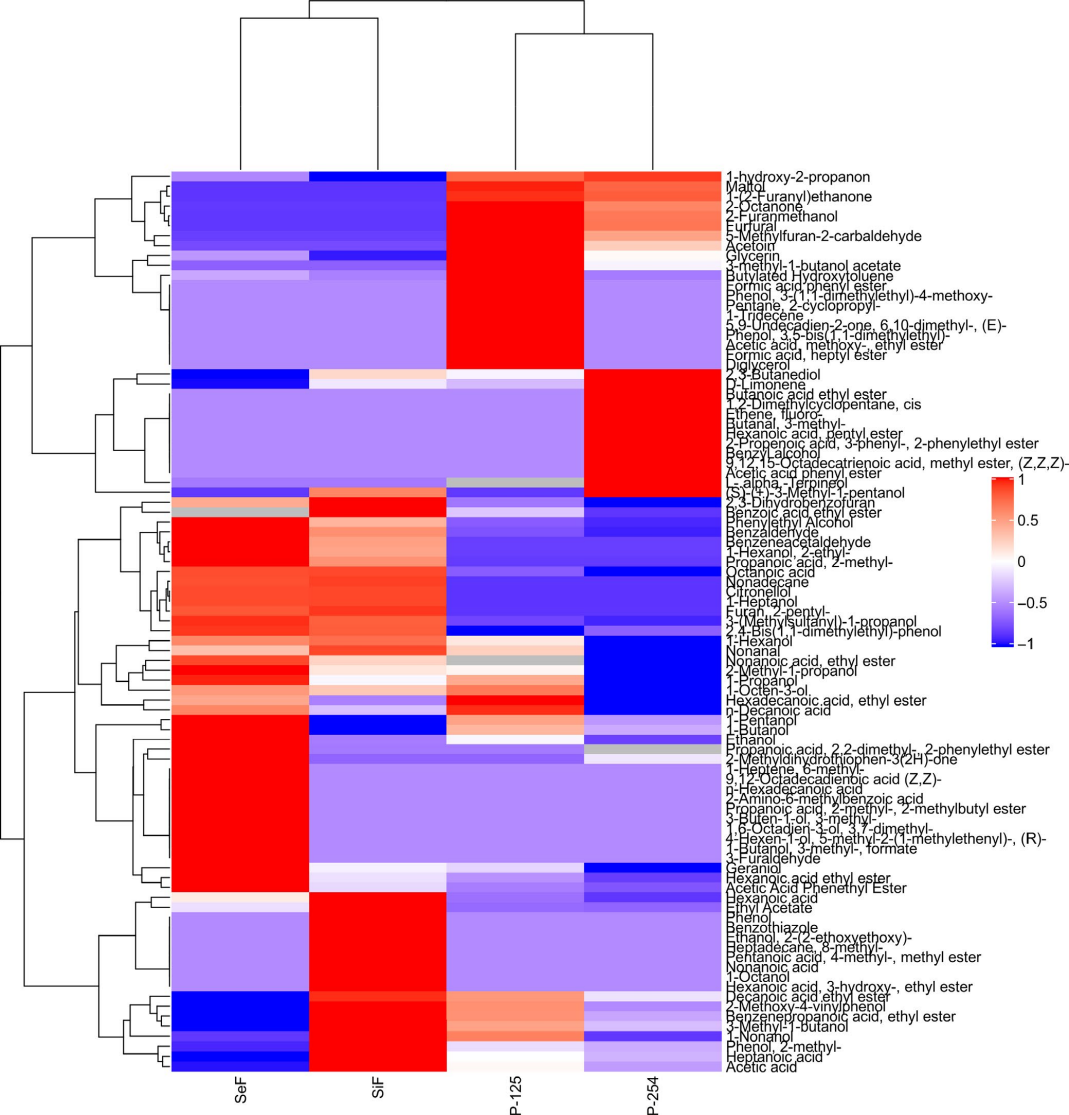
Hierarchical clustering and heat map visualization of volatile compounds of rice wine samples in different fermentations

According to the content of flavoring substances in different fermentation method (Table 2), a cluster heat map was applied to visualize the differences of aroma compounds among different fermentations (Figure 3). The flavoring substances of rice wine with different fermentation methods show different trends in general. Moreover, the aroma compounds were divided into two classes. Class I mainly included acetic esters, fatty acid ethyl esters, higher alcohols, and terpene compounds. Class II mainly contained some kinds of C_6_ compounds, but some kinds of higher alcohols, fatty acid ethyl esters, and other esters were also included. The simultaneous and sequential cofermentations were rich in class I compounds, while pure fermentations were abundant in class II compounds. The results showed that the aroma compound compositions of simultaneous cofermentation were closer to those of sequential cofermentation, which indicated that different inoculation strategies of cofermentations produced wine with different aroma composition profiles. The high content of ethyl ester could make the wine present cheese flavor, fruit flavor, and fatty acids present cream and cheese flavor at low concentration, and sour and rotten flavor will be produced at high concentration (Jolly, Varela, & Pretorius, 2014; Varela et al., 2016). Phenylethanol is a shikimic acid derivative, with rose‐like, bitter, sweet, and peach aromas (Yu et al., 2019); isoamyl alcohol (malt aroma) as a typical representative of grain aroma in wine and the main component of higher alcohols, with apple brandy aroma and pungent taste (Jolly et al., 2014). High content of C_6_ compounds would make wine present pungent and sour taste, while terpene compounds would give wine flower and fruit aroma, and improve the complexity of wine aroma. These results show that sequential cofermentations can improve the quality and sensory of rice wine.

### 3.5 Principal component analysis of rice wine aroma components in different fermentations

**Figure 4:**
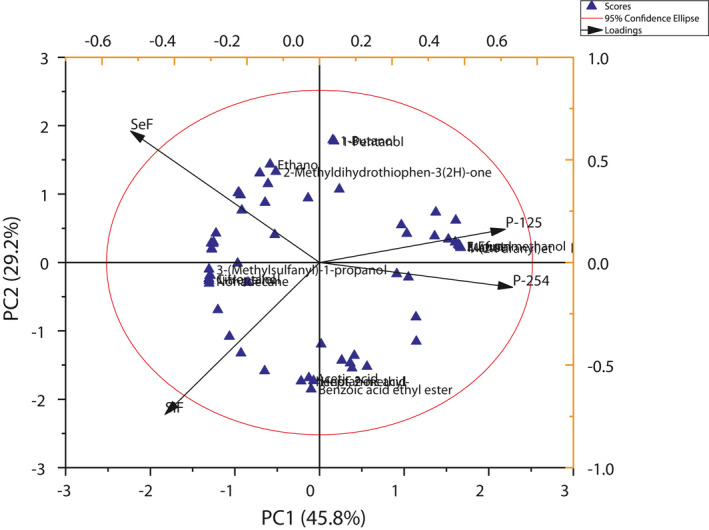
Principal component analysis of varietal volatiles obtained from rice wine in different fermentations

In order to better explain the differences between the biological species and their inoculation sequence on the volatile compounds of rice wine, 62 aroma components (OAV > 1) were selected for principal component analysis (Figure 4). The first two principal components (PC) accounted for 75% of total variance, whereby the first and the second PC, respectively, explained 45.8% and 29.2%. Therefore, the first two principal components can effectively explain variable information. Pure fermentations were positioned in positive PC1 region with higher amounts of 1‐butanol, 1‐pentanol, 2‐furanmethanol, furfural, maltol, and 1‐(2‐furanyl) ethenone, suggesting that pure fermentations are not sufficient to develop complex aroma profiles. On the other hand, simultaneous cofermentation and sequential cofermentation are positioned on the negative part mainly due to their higher levels of acetate esters, ethanol, 1‐heptanol, citronellol, 3‐(methylsulfonyl)‐1‐propanol, benzoic acid ethyl ester, 2‐methyl‐phenol, acetic acid, heptanoic acid, and nonadecane. This is consistent with the results of the above cluster heat map analysis. Interestingly, sequential inoculation was distinguished from co‐inoculation on PC2 (29.2%). However, pure fermentation of *W. anomalus* produces more aroma substances and more harmonious flavor than pure fermentation of *S. cerevisiae*. And cofermentation accounted for significantly higher numbers of volatile compounds indicating production of more complex aroma profiles. These results illustrated that distinctive aroma compound profiles were affected by microbial species and different inoculation strategies. Simultaneous cofermentation rice wine samples would present varietal aroma and low rancidity, whereas sequential cofermentation would take on fruity flavor and rich and mellow fragrance according to their aroma compound composition. The PCA results indicated all rice wine samples were clearly differentiated, indicating that the microbial species and their order of inoculation contributed to different aroma profiles in each sample.

### 3.6 The cumulative odor activity comparison of different volatile chemicals from rice wine samples by different fermentation treatments

**Figure 5:**
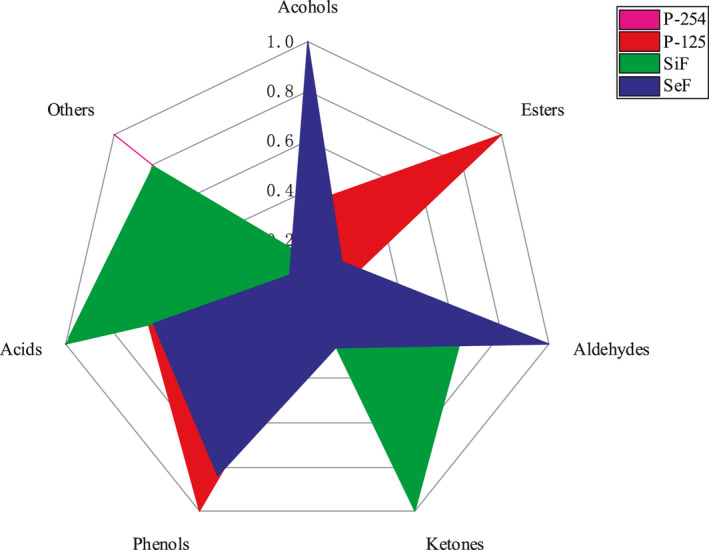
The cumulative odor activity comparison of different volatile chemicals from rice wine samples by different fermentation treatments

The OAV of the volatile components of different chemical categories in the tested rice wine was accumulated, and the data between different fermentation treatments were normalized. The comparison results are shown in Figure 5. Compared with pure fermentation of *S. cerevisiae*, pure fermentation of *W. anomalus* has obvious advantages in the odor activity values of some alcohols and aldehydes, which is related to 1‐butanol, 5‐methylfuran‐2‐carbaldehyde, maltol, and other substances. Fatty taste at low concentration is beneficial to increase the complexity of aroma, while pure fermentation of *S. cerevisiae* isoamyl alcohol, 3‐methylbutanal, and other substances has low odor activity. Mixed fermentation improves the odor activity of fermented aroma components in rice wine. Simultaneous cofermentation has obvious advantages in the odor activity of ketones and acids, which is related to decanoic acid and heptanoic acid. Furthermore, there is higher 1‐hexanol, 1‐octanol odor activity. Sequential cofermentation improves the odor activity of alcohols, phenols, and aldehydes, especially terpenoids, but decreases in esters. This was related to phenylethyl alcohol, 1‐propanol, 2‐methyl‐1‐propanol, 2‐ethyl‐1‐hexanol, citronellol, and other substances. According to the aroma characteristics of various odor components in rice wine fermentation (Yang et al., 2019; Yu et al., 2019), pure fermentation of *S. cerevisiae* rice wine sample has a better fatty and herbaceous aroma and pure fermentation of *W. anomalus* rice wine sample has richer fruit aroma and caramel aroma. Sequential cofermentation rice wine sample has the highest fermented fruit flavor. Due to the cumulative effect of different esters on the aroma perception, the fruit aroma of rice wine comes from the effect of mixed esters. But the intensity of fruit aroma perception is not directly proportional to the total ester content, and it is related to the special ratio, which explains the reason that although the sequential cofermentation yield of ester is not high, the fruit aroma in the fermentation broth is strong. Simultaneous cofermentation also has a strong fatty and herbal aroma, and sequential cofermentation has a strong mellow and cereal aroma. The overall aroma is more coordinated and flower astringency. Therefore, the selection of suitable yeast and fermentation methods for wine production could be a promising way to regulate the characteristics of rice wine.

## 4 CONCLUSIONS

The growth of *W. anomalus* was suppressed by the presence of *S. cerevisiae* produced. Rice wines produced with cofermentations of *W. anomalus* and *S. cerevisiae* had lower volatile acidity, more kinds of flavor compounds, and fermentative aroma contents. Moreover, the sequential cofermentation was more conducive to improve rice wine flavor and quality than the simultaneous cofermentation, due to its reduced thiols, increased such fermentative compounds as higher alcohols, esters, phenylethyls, and terpenes. And the sequential cofermentation had better effect on regulating the odor activity of the mellow and cereal flavor components and has a better coordination on the overall flavor of rice wine. In general, the selection of suitable yeast and fermentation methods for rice wine production was important to improve rice wine quality. The sequential cofermentation with *S. cerevisiae* and *W. anomalus* was an available method to produce rice wine with good flavor. The results of this study would provide a guidance for mixed fermentation of other *non‐Saccharomyces* yeast in rice wine brewing.

## CONFLICT OF INTEREST

The authors declare that they have no conflict of interests.

## AUTHOR CONTRIBUTIONS

Lihua Chen designed the research, collected test data, and wrote the original draft. Dongna Li designed the research, collected test data, wrote the original draft, and involved in formal analysis. Lixia Ren collected test data. Xia Ma reviewed and edited the manuscript. Shiqing Song reviewed and edited the manuscript. Yuzhi Rong, designed the research, involved in formal analysis, and wrote, reviewed, and edited the manuscript.
